# Dr. Austin D. Smith

**Published:** 1904-05

**Authors:** 


Dr. Austin D. Smith, of East Pembroke, N. Y., died of apoplexy at his home, March 25, 1904, aged 74 years. He graduated at Geneva Medical College in 1869, practised first at Alexander, then at Aurora, Akron, and Oakfield, finally locating at East Pembroke in 1875. He is survived by his wife, one son, Dr. Elliott C. Smith, of Corfu, and a daughter.

